# Prognostic Significance of *SLFN11* Methylation in Plasma Cell-Free DNA in Advanced High-Grade Serous Ovarian Cancer

**DOI:** 10.3390/cancers14010004

**Published:** 2021-12-21

**Authors:** Victoria Tserpeli, Dimitra Stergiopoulou, Dora Londra, Lydia Giannopoulou, Paul Buderath, Ioanna Balgkouranidou, Nikolaos Xenidis, Christina Grech, Eva Obermayr, Robert Zeillinger, Kitty Pavlakis, Theodoros Rampias, Stylianos Kakolyris, Sabine Kasimir-Bauer, Evi S. Lianidou

**Affiliations:** 1 Analysis of Circulating Tumor Cells, Lab of Analytical Chemistry, Department of Chemistry, National and Kapodistrian University of Athens, 15771 Athens, Greece; victserp@chem.uoa.gr (V.T.); dimitrasterg@chem.uoa.gr (D.S.); doralo@chem.uoa.gr (D.L.); giannop@chem.uoa.gr (L.G.); 2 Department of Gynecology and Obstetrics, University Hospital of Essen, University of Duisburg-Essen, Hufelandstrasse 55, D-45122 Essen, Germany; paul.buderath@uk-essen.de (P.B.); Sabine.Kasimir-bauer@uk-essen.de (S.K.-B.); 3 Department of Oncology, Medical School, Democritus University of Thrace, 68100 Alexandroupolis, Greece; ibalgkou@med.duth.gr (I.B.); nxenidis@med.duth.gr (N.X.); skakolyr@med.duth.gr (S.K.); 4 Department of Obstetrics and Gynecology, Medical University of Vienna, 1090 Vienna, Austria; christina.grech@meduniwien.ac.at (C.G.); eva.obermayr@meduniwien.ac.at (E.O.); robert.zeillinger@meduniwien.ac.at (R.Z.); 5 Pathology Department, IASO Women’s Hospital, 15123 Athens, Greece; epavlaki@med.uoa.gr; 6 Basic Research Center, Biomedical Research Foundation of the Academy of Athens, 11527 Athens, Greece; trampias@bioacademy.gr

**Keywords:** liquid biopsy, plasma cell-free DNA, Schlaffen11, DNA methylation, high-grade serous ovarian cancer, prognostic biomarker, progression free survival, methylation specific PCR

## Abstract

**Simple Summary:**

In the present study, we examined the methylation status of six gene promoters (*BRCA1*, *CST6*, *MGMT*, *RASSF10*, *SLFN11* and *USP44)* in high-grade serous ovarian cancer (HGSOC). We evaluated the prognostic significance of DNA methylation of these six gene promoters in primary tumors (FFPEs) and plasma cfDNA samples from patients with early, advanced and metastatic HGSOC. We report for the first time that the DNA methylation of *SLFN11* in plasma cfDNA was significantly correlated with worse PFS (*p* = 0.045) in advanced stage HGSOC. Our results strongly indicate that *SLFN11* epigenetic inactivation could serve as a potential prognostic DNA methylation biomarker and a predictor of resistance to platinum-based chemotherapy in ovarian cancer.

**Abstract:**

Background: Epigenetic alterations in ctDNA are highly promising as a source of novel potential liquid biopsy biomarkers and comprise a very promising liquid biopsy approach in ovarian cancer, for early diagnosis, prognosis and response to treatment. Methods: In the present study, we examined the methylation status of six gene promoters (*BRCA1*, *CST6*, *MGMT*, *RASSF10*, *SLFN11* and *USP44)* in high-grade serous ovarian cancer (HGSOC). We evaluated the prognostic significance of DNA methylation of these six gene promoters in primary tumors (FFPEs) and plasma cfDNA samples from patients with early, advanced and metastatic HGSOC. Results: We report for the first time that the DNA methylation of *SLFN11* in plasma cfDNA was significantly correlated with worse PFS (*p* = 0.045) in advanced stage HGSOC. Conclusions: Our results strongly indicate that *SLFN11* epigenetic inactivation could be a predictor of resistance to platinum drugs in ovarian cancer. Our results should be further validated in studies based on a larger cohort of patients, in order to further explore whether the DNA methylation of *SLFN11* promoter could serve as a potential prognostic DNA methylation biomarker and a predictor of resistance to platinum-based chemotherapy in ovarian cancer.

## 1. Introduction

Ovarian cancer still remains the most lethal female cancer since, in most cases, it is diagnosed at an advanced stage. Epithelial ovarian cancer (EOC) represents the main type and is characterized by histological and molecular heterogeneity. Primary therapy for patients with EOC is radical debulking surgery, followed by adjuvant platinum- and paclitaxel-based chemotherapy [1,2]. The addition of the anti-vascular endothelial growth factor (VEGF) antibody bevacizumab to adjuvant chemotherapy has been shown to prolong disease-free survival in patients with advanced EOC in two randomized controlled phase III trials (GOG-0218 and ICON7) [3,4]. In most cases, patients respond initially to primary treatment; nevertheless, the large majority of them will experience chemo-resistance and, finally, recurrent disease. Hence, overall survival is generally low [5,6]. The most common histotype of EOC is high-grade serous ovarian cancer (HGSOC), a highly aggressive disease that is often diagnosed at an advanced FIGO stage [7,8]. A few innovative targeted therapies are now available for ovarian cancer, including the anti-angiogenetic antibody bevacizumab [3] and the PARP inhibitors olaparib, rucaparib and niraparib, which are FDA-approved as maintenance treatments for platinum-sensitive recurrent ovarian cancer patients harboring a germline or somatic *BRCA1/2* mutation (in the case of olaparib) while independent of *BRCA* status in the cases of rucaparib and niraparib [9,10,11,12,13].

Epigenetic modifications hold an important role in cancer initiation and progression; DNA methylation at specific sites of a gene, mainly at CpG islands in the promoter region, has a direct effect on gene expression [14,15,16]. Alterations in DNA methylation patterns are frequently observed in EOC, and it is also argued that each histological subtype is characterized by different methylation motifs [17]. Additionally, more and more studies indicate the role and clinical potential of aberrant DNA methylation as an ovarian cancer biomarker for early diagnosis, prognosis and response to treatment [18,19,20,21].

Liquid biopsy, a minimally invasive blood-based approach, provides the potential of monitoring a tumor’s evolution in real time and offers novel insights on early cancer diagnosis and treatment [22]. The major components of liquid biopsy are circulating tumor cells (CTCs), circulating tumor DNA (ctDNA), circulating cell-free microRNAs (cfmiRNAs) and circulating extracellular vesicles (EVs), which are shed from primary tumors or metastatic sites into peripheral blood [23]. Liquid biopsy has generated enormous interest as a valuable source of biomarkers on prognosis as well as response to treatment and holds a strong potential for novel approaches in the therapeutic management of cancer [24].

A tiny subgroup of cell-free DNA (cfDNA) that is shed in circulation by cancer cells is called ctDNA and, therefore, carries most of the genetic and epigenetic modifications identified in the primary tumors [25]. Epigenetic alterations in ctDNA are highly promising as a source of novel potential liquid biopsy biomarkers and comprise a very promising liquid biopsy approach [26] for the early diagnosis [27], prognosis and response to treatment of ovarian cancer since they are considered an easily acceptable source of tumor DNA [28].

DNA methylation analysis in CTCs and ctDNA has strong potential to provide a valuable source of novel circulating epigenetic biomarkers for diagnosis, prognosis, risk assessment and disease monitoring in many types of cancer [29]. Our group was the first to demonstrate epigenetic alterations in CTCs and the corresponding ctDNA [30,31,32]. More specifically, we have shown the prognostic significance of *SOX17* [31], *CST6* [32] and *BRMS1* [33] methylation in ctDNA and demonstrated the prognostic significance of *ESR1* methylation in CTCs in patients with breast cancer [34]. In HGSOC, we have recently reported that the methylation of *RASSFIA* [35] and *ESR1* [36] in ctDNA provide prognostic information.

In the present study, we examined the methylation status of six gene promoters (*BRCA1*, *CST6*, *MGMT*, *RASSF10*, *SLFN11* and *USP44)* in HGSOC. All of these genes play an important role in the biology of ovarian cancer. More specifically, *BRCA1* and *MGMT* are both DNA repair genes [37,38]; *RASSF10* and *USP44* are involved in cell cycle regulation [39,40]. *SLFN11* is an inhibitor of DNA replication that promotes cell death in response to DNA damage [41], and *CST6* directly inhibits the activity of cysteine-type endopeptidases [42]. The DNA methylation of *BRCA1* [43] and *MGMT* [44] has been previously reported in ovarian cancer. Epigenetic inactivation of the putative DNA/RNA helicase *SLFN11* in human cancer has been reported to confer resistance to platinum drugs [45]. We have chosen to evaluate the DNA methylation of *CST6*, *RASSF10* and *USP44* in our study, even if these genes have not been studied so far in ovarian cancer, based on the tumor suppressive functions reported for these genes in other types of cancer [46,47,48].

The aim of our study was to evaluate the prognostic significance of the DNA methylation of these six gene promoters in primary tumors (FFPEs) and plasma cfDNA samples from patients with early, advanced and metastatic HGSOC.

## 2. Material and Methods

### 2.1. Clinical Samples

Our study material consisted of three groups of patients with HGSOC, according to their FIGO stage; (a) *early stage HGSOC (FIGO I-II)*, *group A*: consisting of 55 primary ovarian tumors (formalin-fixed paraffin-embedded tissues, FFPEs), (b) *advanced stage HGSOC (FIGO III)*, *group B*: consisting of 62 primary ovarian tumor FFPEs and 84 plasma cfDNA samples (2 mL), in which 40 of the corresponding primary tumor were available and (c) *metastatic stage HGSOC (FIGO IV)*, *group C*: consisting of 49 plasma cfDNA samples. The clinicopathological features for each of these three groups are shown in Table 1. Two groups of non-cancerous samples were used as controls: (a) a group of 16 normal fallopian tubes’ FFPEs that were obtained from women at a reproductive age and (b) a group of 27 plasma samples obtained from healthy donors (HD). The normal fallopian tube samples and 66 FFPEs (50 from patients with early stage HGSOC and 16 with advanced HGSOC) were obtained from the Pathology Department of IASO Women’s Hospital in Athens, Greece. According to the protocol used in their Pathology Department, the whole fimbria is processed together with four sections from the body of the tube. Since serous tubal intraepithelial carcinomas and their invasive counterparts are reported as arising in fimbria, we believe that by using the above protocol we nearly eliminate the possibility of missing an early carcinoma. A quantity of 53 FFPE tissue blocks (6 from patients with early stage HGSOC and 47 with advanced HGSOC from patients) were retrieved from the Institute of Pathology and Neuropathology of the University Hospital of Essen, Essen, Germany. All of these blocks and FFPE sections were stained with hematoxylin and eosin and were prepared and reviewed by a pathologist. We only included patients who had at least a greatest possible intra-tissue tumor content of 60%. According to our pathologists, these were reasonable amounts of tumor tissue to study since 100% purity of tumor tissue can only be achieved in rare cases. Finally, all plasma samples were obtained from the Department of Pathology and the Department of Gynecology and Obstetrics, University Hospital of Essen, University of Duisburg-Essen, Essen, Germany; the Department of Obstetrics and Gynecology, Medical University of Vienna, Vienna, Austria; and the Department of Medical Oncology of University General Hospital of Alexandroupolis, Alexandroupolis, Greece. All patients gave their informed written consent to participate in the study, which was approved by the Local Essen Research Ethics Committee (16-6916-BO; 17-7859-BO), IASO Women’s Hospital Ethics Committee (Date: 05/2014) and General University Hospital of Alexandroupolis Ethics Committee (Date: 25/06/2020). For the plasma samples, approval came from the University of Vienna Ethics Committee (EK 366/2003).

### 2.2. DNA Isolation from FFPEs and Plasma

Genomic DNA (gDNA) was isolated from FFPEs using the QIAamp^®^ DNA FFPE Tissue Kit 50 (QIAGEN^®^, Hilden, Germany), according to manufacturer instructions. For the plasma, 2 × 5 mL peripheral blood samples were collected in EDTA tubes at the time point of diagnosis, before tumor surgery and before the application of therapeutic substances with an S-Monovette. Blood was centrifuged at 1500× *g* for 10 min, and the plasma was stored at −80 °C until further analysis. Additionally, cfDNA was extracted from plasma (2 mL) using the QIAamp^®^ Circulating Nucleic Acid kit 50 (QIAGEN^®^, Hilden, Germany), according to the manufacturer’s instructions. The DNA concentration was determined in the Nanodrop ND-1000 spectrophotometer (Thermo Fisher Scientific, Inc., Waltham, MA, USA), as previously described [35].

### 2.3. Sodium Bisulfite Conversion

Up to 0.5 μg of plasma cfDNA and 1 μg of gDNA and were chemically modified with sodium bisulfite (SB), in order to convert only the non-methylated cytosines to uracils and not the methylated ones. SB conversion was performed with the EZ DNA Methylation-Gold^TM^ Kit 200 (ZYMO Research, Irvine, CA, USA), according to the manufacturer’s instructions, as previously described [35]. The Universal Methylated Human DNA Standard (ZYMO Research, Irvine, CA, USA) was used as the 100% methylated control. In each conversion reaction, the dH_2_O and gDNA from the 100% methylated control were used as negative and positive controls, respectively. To evaluate the quality of the SB converted DNA in our samples, we applied a specific MSP assay for *ACTB*, as previously described [34]. Real-time PCR amplification occurred in all of the SB converted DNA samples. The SB converted DNA was stored at −70 °C until use.

### 2.4. Real-Time Methylation-Specific PCR (Real-Time MSP)

All of the real-time MSP assays developed and used in this study are highly specific and sensitive, as they detect down to 0.1% of the methylated sequences in the presence of 99.9% non-methylated sequences. The developed real-time MSP assays are not quantitative, hence we do not use a cutoff, and we report a sample as positive when we detect an amplification signal and as negative when no amplification is observed. All of the real-time MSP reactions were performed in the cobas^®^ z480 and LightCycler^®^ 2.0 instruments (Roche Diagnostics, Mannheim, Germany), and the final reaction volume was 10 μL. In the Methylated Human DNA Standard (ZYMO Research, Irvine, CA, USA), 100% methylated DNA was used as the positive control for all of the real-time MSP reactions. The human placental DNA (Sigma-Aldrich, Burlington, MA, USA) was used as the non-methylated control for the *BRCA1*, *CST6*, *RASSF10* and *USP44* real-time MSP assays. This non-methylated DNA control was mixed with the 100% methylated standard for the preparation of serial dilutions of known concentrations (0.1%, 1%, 10%, 30% and 50%) for the evaluation of the analytical sensitivity for all of the MSP assays. The placental DNA was found methylated for *MGMT* and *SLFN11*, so a pool of healthy donor DNA samples was used as a fully non-methylated control in the real-time MSP assays. The same pool was also used for the preparation of serial dilutions, as mentioned above.

For the detection of *RASSF10* methylation, we first in silico designed the MSP primers and further developed and validated a highly specific and sensitive real-time MSP assay. To begin, 1 μL of SB converted DNA was added in the PCR reaction mix, which consisted of 1× PCR buffer (Promega, Madison, WI, USA), 2.5 mM MgCl_2_ (Promega, Madison, WI, USA)_,_ 0.25 μΜ of each dNTP (Invitrogen, Carlsbad, CA, USA), 0.15 μg/μL BSA (Sigma-Aldrich, Burlington, MA, USA), 0.25 μΜ of each primer (Integrated DNA Technologies, Coralville, IA, USA), 1× LC Green^®^ (Idaho Technology, Salt Lake City, UT, USA) and 0.05 U/μL GoTaq^®^ DNA polymerase (Promega, Madison, WI, USA). Then, dH_2_O was added to the final volume of 10 μL. Protocol conditions were: 1 cycle at 95 °C for 2 min, followed by 45 cycles of 95 °C for 10 s, 65 °C for 20 s and 72 °C for 20 s. A melting curve analysis was performed next: 55 °C for 20 s, 95 °C continuous, 95 °C for 1 min and a final cooling cycle at 40 °C for 30 s.

For the detection of *SLFN11* methylation, we first in silico designed the MSP primers and further developed and validated a highly specific and sensitive real-time MSP assay. To begin, 1 μL of SB converted DNA was added in the PCR reaction mix, which consisted of 1× PCR buffer (Promega, Madison, WI, USA), 2 mM MgCl_2_ (Promega, Madison, WI, USA), 0.15 mM of each dNTP (Invitrogen, Carlsbad, CA, USA), 0.15 μg/μL BSA (Sigma-Aldrich, Burlington, MA, USA), 0.2 μM of each primer (Integrated DNA Technologies, Coralville, IA, USA), 1× LC Green^®^ (Idaho Technology, Salt Lake City, UT, USA) and 0.05 U/μL GoTaq® DNA polymerase (Promega, Madison, WI, USA). Then, dH_2_O was added to a final volume of 10 μL. Protocol conditions were: 1 cycle at 95 °C for 2 min, followed by 45 cycles of 95 °C for 10 s, 65 °C for 20 s and 72 °C for 20 s. A melting curve analysis was performed next: 55 °C for 10 s, 95 °C continuous, 95 °C for 1 min and a final cooling cycle at 40 °C for 30 s.

For the detection of *BRCA1* and *MGMT* methylation, we used the MSP primers from previous studies [49,50] after performing all of the optimization steps for real-time MSP; for the detection of *CST6* and *USP44* methylation, we used the real-time MSP assays that we previously developed and validated [46,48]. In Table 2, the real-time MSP primer sequences of each gene are given.

### 2.5. Statistical Analysis

Τhe concordance between the DNA methylation of each gene in primary tumors and paired plasma cfDNA was calculated using Pearson’s χ^2^ and Cohen’s kappa coefficient. Pearson’s χ^2^ and Fischer’s exact test were used for the estimation of the correlation between the methylation status for each gene and the patients’ clinicopathological characteristics (Table 1), and *p*-values < 0.05 were considered as statistically significant. The k values were interpreted according to the guidelines. The Kaplan–Meier method was used for the calculation of the overall survival (OS) and progression-free survival (PFS) curves and a log-rank test was performed for the comparisons. All statistical analysis was performed by using the SPSS version 26.0 (IBM^®^ SPSS^®^ Statistics).

## 3. Results

**Figure 1:**
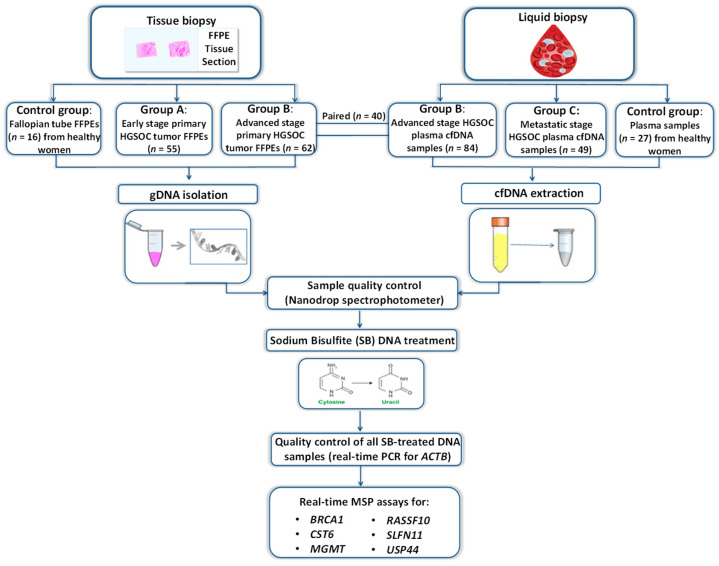
Schematic flowchart of the study.

A schematic flowchart of the study is shown in Figure 1.

### 3.1. DNA Methylation Markers in HGSOC Primary Tumors and Plasma cfDNA

The methylation status of each gene was first investigated in a control group of 16 fallopian tube samples (FFPEs) from healthy individuals and 27 plasma samples from HD. All 16 fallopian tube samples were found negative for *MGMT*, *RASSF10*, *SLFN11* and *USP44* methylation 0/16 (0.0%), while *CST6* methylation was detected in 1/16 (6.3%) and *BRCA1* methylation in 4/16 (25.0%) of them. DNA methylation was not detected for any of the tested genes (0/27, 0.0%) in the 27 HD plasma cfDNA samples.

The DNA methylation of all of these six gene promoters was further tested by real-time MSP in all of the primary tumor samples. In plasma cfDNA of advanced stage (group B) and metastastic stage (group C), we investigated the DNA methylation status only for the genes that were found methylated in the primary tumors of the advanced stage group B at percentages higher than 10.0% (*BRCA1*, *SLFN11* and *USP44)***.** Finally, we evaluated the prognostic significance of each individual DNA methylation marker in the group of patients for whom their clinicopathological characteristics were available (advanced stage groups and metastatic group).

**Figure 2:**
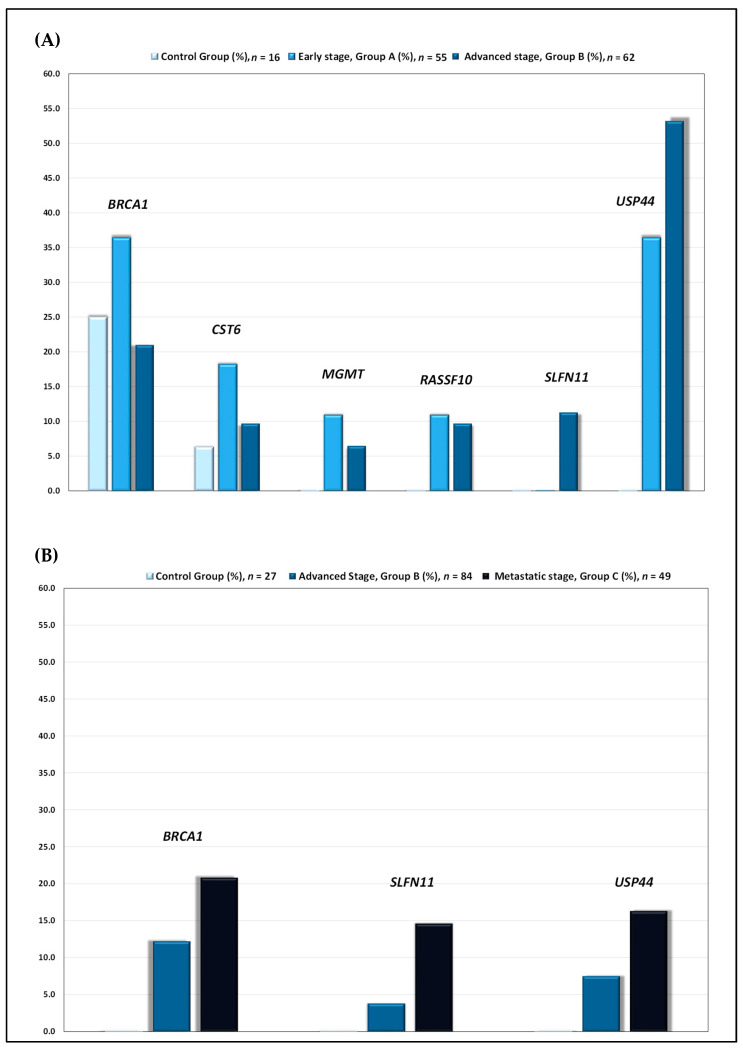
(**A**) DNA methylation of *BRCA1*, *CST6*, *MGMT*, *RASSF10*, *SLFN11* and *USP44* gene promoters in primary tumors of patients diagnosed with early (FIGO I-II) and advanced stage (FIGO III) HGSOC. (**B**) DNA methylation of *BRCA1*, *SLFN11* and *USP44* gene promoters in plasma cfDNA of patients diagnosed with advanced (FIGO III) and metastatic stage (FIGO IV) HGSOC.

Early stage (group A): In this group, in primary tumors, DNA methylation was detected as follows: *BRCA1* in 20/55 (36.4%), *CST6* in 10/55 (18.2%), *MGMT* in 6/55 (10.9%), *RASSF10* in 6/55 (10.9%) and *USP44* in 20/55 (36.4%). No methylation was detected for *SLFN11* promoter (0.0%) (Figure 2A).

Advanced stage (group B): In this group, in primary tumors, DNA methylation was detected as follows: *BRCA1* in 13/62 (21.0%), *CST6* in 6/62 (9.7%), *MGMT* in 4/62 (6.5%), *RASSF10* in 6/62 (9.7%), *SLFN11* in 7/60 (11.3%) and *USP44* in 33/60 (53.2%) (Figure 2A). In plasma cfDNA, methylation was detected as follows: *BRCA1* in 10/82 (12.2%), *SLFN11* in 3/80 (3.8%) and *USP44* in 6/80 (7.5%) (Figure 2B).

Metastatic stage (group C): In plasma cfDNA, *BRCA1* methylation was detected in 10/48 (20.8%), *SLFN11* methylation in 7/48 (14.6%) and *USP44* methylation in 8/49 (16.3%) (Figure 2B)

### 3.2. Evaluation of the Prognostic Significance of the DNA Methylation of BRCA1, SLFN11 and USP44 in HGSOC

**Figure 3:**
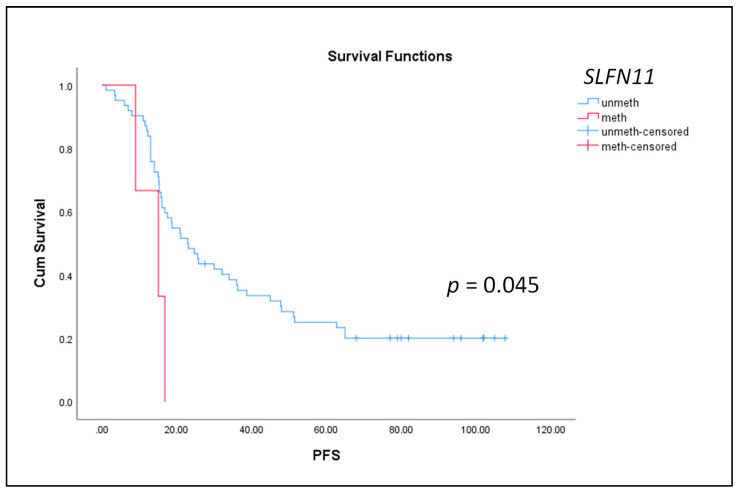
Kaplan–Meier estimates of patients with advanced HGSOC (FIGO III) in relation to DNA methylation of *SLFN11* in plasma cfDNA and PFS (*n* = 84).

We further evaluated the prognostic significance of the DNA methylation of *BRCA1*, *SLFN11* and *USP44* in patients in the advanced and metastatic group (group B and C, respectively). In group B, the median OS was 41 months, and the median PFS was 22 months, while in group C, the median OS was 44 months, and the median PFS was 19 months. A Kaplan–Meier analysis was performed to estimate the correlation between OS and PFS with the detection of the DNA methylation of each of the six genes tested. Our results indicated that only *SLFN11* methylation in plasma cfDNA in the advanced group (FIGO III) was significantly correlated with worse PFS (*p* = 0.045, log-rank test) (Figure 3). No significant correlations were observed among OS, PFS and *BRCA1* and *USP44* promoter methylation, neither in the advanced stage nor in the metastatic stage (data not shown).

### 3.3. Concordance of DNA Methylation between Primary Tumors and Corresponding Plasma cfDNA in Advanced Stage HGSOC

We directly compared the DNA methylation of *BRCA1*, *SLFN11* and *USP44* in the primary tumors and corresponding plasma cfDNA in samples from group B patients. In most cases, there was a weak agreement between primary tumors and paired plasma samples (Table 3 and Table 4) but mostly for negative samples; more specifically, the concordance was as follows: *BRCA1*: 31/40 (77.5%), *SLFN11*: 32/37 (86.5%) and *USP44*: 21/37 (56.8%). According to the guidelines for the interpretation of Cohen’s kappa values, for *BRCA1* (*p* = 0.573, k = −0.125), *SLFN11* (*p* = 0.892, k = −0.045) and *USP44* (*p* = 0.230, k = 0.114), there is a very weak agreement in the DNA methylation of these three genes between the primary tumors and corresponding plasma cfDNA.

## 4. Discussion

In the current study, we evaluated the prognostic significance of *BRCA1*, *CST6*, *MGMT*, *RASSF10*, *SLFN11* and *USP44* promoter methylation in primary tumors and plasma cell-free DNA in early, advanced and metastatic HGSOC, using novel highly specific and sensitive real-time MSP assays.

Recently, there has been an increasing need to discover new ovarian cancer biomarkers that could potentially contribute to early detection and progression of this disease. Epigenetic alterations occur early in cancer development and are now intensively evaluated as highly promising biomarkers. More and more studies indicate the role and clinical potential of aberrant DNA methylation in ovarian cancer for early diagnosis, prognosis and response to treatment [18,19,20,21]. Moreover, the detection of epigenetic markers in plasma cfDNA provides a real-time monitoring of tumor progression and is a unique and minimally invasive way to monitor the response to anticancer therapies [28].

We selected these six genes to study based on the importance of their role in the biology of ovarian cancer. We detected DNA methylation in all of these gene promoters at different frequencies in the three HGSOC groups tested. According to our results, only the aberrant methylation of *SLFN11* in plasma-derived cfDNA was correlated to worse PFS in advanced stage HGSOC. This finding is in agreement with a previous study which showed that patients with ovarian and non-small cell lung cancer carrying *SLFN11* hypermethylation in primary tumors had a poor response to both cisplatin and carboplatin treatments [45]. According to the literature, variable levels of *BRCA1* promoter methylation in ovarian cancer have been previously reported, ranging from 5–90% [51,52,53]. In another study, Wang et al. reported that compared to stage I and healthy subjects, there were higher *BRCA1* promoter methylation frequencies in stage II and III ovarian cancers [54]. In the case of *MGMT* promoter methylation, a recent meta-analysis that evaluated the samples of 10 ovarian cancer studies showed the importance of *MGMT* methylation in ovarian cancers, but the results are controversial with a variation in methylation frequency ranges [44]. The association of *MGMT* methylation with certain histological types was previously observed [55]. *MGMT* hypermethylation was described as common in most histological subtypes, with the exception of serous carcinoma [56]. Our results are in concordance with the reported findings that *MGMT* methylation is detected at a low occurrence in serous carcinoma. The discrepancies between the *BRCA1* and *MGMT* methylation frequencies reported in our study and the above studies could possibly be explained by the fact that different primers for MSP, designed at different CpG sites, were used.

Additionally, we observed that the methylation frequencies for all of the genes studied were higher in the primary tumors of the patients at an early stage (group A) when compared to the primary tumors of the patients at an advanced stage (group B), whereas the plasma cfDNA methylation frequencies for all of the genes studied were higher in the plasma cfDNA of the patients with verified metastasis (group C) when compared to the plasma cfDNA of the patients at an advanced stage (group B). We observed a weak agreement in the DNA methylation of of *BRCA1*, *SLFN11* and *USP44* promoter methylation in the primary tumors and corresponding plasma cfDNA in our group of patients with advanced stage HGSOC. This observation could probably be explained on the basis of tumor heterogeneity; tissue biopsy represents a snapshot of a tumor’s molecular characteristics, while in plasma cfDNA, we detect genetic and epigenetic features of a tumor’s DNA, shed at a specific time point from different metastatic sources in plasma. Moreover, circulating tumor DNA in plasma is derived mainly from circulating tumor cells and apoptotic cancer cells, and it is only a small fraction of plasma cfDNA. Thus, this discrepancy could be due to a low concentration of ctDNA which could not be detected by MSP.

Only a few reports up to now have examined concurrent methylation of multiple genes in association with their prognostic significance in ovarian cancer. Su et al. reported that the DNA methylation of *SFRP1 SFRP2*, *SFRP1*, *SFRP2*, *SOX1* and *LMX1A* correlated with recurrence and worse OS in ovarian cancer [57]. In another study, Lin et al. investigated the *CDH1*, *DLEC1* and *SFRP5* gene methylation panel for advanced EOC and demonstrated that the detection of DNA methylation in two or all three of these genes was a significant marker of recurrence and OS [58]. Montavon et al. evaluated the methylation patterns of ten genes (*BRCA1*, *EN1*, *DLEC1*, *HOXA9*, *RASSF1A*, *GATA4*, *GATA5*, *HSULF1*, *CDH1* and *SFN*) in HGSOC and used 12 benign ovarian surface epithelium (OSE) samples as a control group; they reported a high variation in the DNA promoter methylation for all of these genes in the primary tumors and that *HOXA9* methylation was observed in 95% of cases. Furthermore, it was shown that the DNA methylation of *DLEC1* promoter was associated with disease recurrence in primary tumors, but these markers were not evaluated in plasma cfDNA [59]. We have previously reported that the detection of *RASSF1A* and *ESR1* promoter methylation in plasma cfDNA provides prognostic information in the patients with advanced stage HGSOC (group B) [35,36].

We report for the first time that the DNA methylation of *SLFN11* in plasma cfDNA was significantly correlated with worse PFS in advanced stage HGSOC. Our results strongly indicate that *SLFN11* epigenetic inactivation could be a predictor of resistance to platinum drugs in ovarian cancer. Recent functional studies on cancer cell lines have identified *SLFN11* as the strongest predictor of sensitivity to DNA-damaging agents (DDAs), including platinum-based chemotherapy [60]. Very recently, Winkler et al. have shown that in high-grade serous ovarian carcinoma specimens before platinum-based chemotherapy treatment, the *SLFN11* density at the protein level in both the neoplastic and microenvironmental components was independently associated with a favorable outcome and that *SLFN11* expression in both infiltrating innate and adaptive immune cells was associated with immune activation in HGSOC [61]. Our finding of a lack of *SLFN11* expression due to epigenetic inactivation through DNA methylation are, in fact, verifying the findings by Winkler et al. [61].

## 5. Conclusions

We report for the first time that the DNA methylation of *SLFN11* in plasma cfDNA was significantly correlated with worse PFS in advanced stage HGSOC. Our results strongly indicate that *SLFN11* epigenetic inactivation could be a predictor of resistance to platinum drugs in ovarian cancer. Our results should be further validated in studies based on a larger cohort of patients, in order to explore to a greater extent whether the DNA methylation of *SLFN11* promoter could serve as a potential prognostic DNA methylation biomarker and a predictor of resistance to platinum-based chemotherapy in ovarian cancer.

## Figures and Tables

**Table 1:** Clinicopathological characteristics of HGSOC patients.

Clinicopathological Characteristics	Group A Early, (FIGO I–II) *n* = 55, (*n*%)	Group B Advanced (FIGO III) *n* = 104, (*n*%)	Group C Metastatic (FIGO IV) *n* = 49, (*n*%)
**Histology**			
Serous	55 (100.0)	104 (100.0)	49 (100.0)
**Tumor grade (G)**			
G1	-	-	-
G2	4 (7.3)	22 (21.2)	4 (8.2)
G3	51 (100.0)	82 (78.9)	45 (91.8)
**FIGO stage**			
I	14 (25.5)		-
II	31(56.5)		-
III	-	104 (100.0)	-
IV	-		49 (100.0)
**Age**	**Median age = 57**	**Median** **age = 66**	**Median age = 61**
≥median age	28 (50.9)	52 (50.0)	24 (49.0)
<median age	27 (49.1)	52 (50.0)	25 (51.0)
**Distant metastasis (M)**			
M0	55 (100.0)	104 (100.0)	-
M1	-	-	49 (100.0)
Unknown	-	-	-

**Table 2:** Primer and probe sequences used in real-time methylation-specific PCR assays.

Gene	Primer Sequences 5′-3′ (For SB–Converted Sequences)	Detection	PCR Product, bp
*BRCA1*	F: TCGTGGTAACGGAAAAGCGC R: AAATCTCAACGAACTCACGCCG	LC green	75
*CST6*	F: TCGAGTTTCGTTTTATTTTAGGTC R: CATAACCGTCAATACCGTCG	FAM–TAGCGGGTAAAAGTGCGCGGTCTAAGTT–BBQ	134
*MGMT*	F: TTTCGACGTTCGTAGGTTTTCGC R: CGTTTCGTTTTCGGAAGAGTGC	LC green	81
*RASSF10*	F: CGTCGTTTTAGTAGATTTCGGTC R: CGTCGAAACAAATAATACGACG	LC green	143
*SLFN11*	F: ATGGAGGGAGCGAGGAGAC R: AACGAATCTACTAAACCCGCG	LC green	155
*USP44*	F: TATTTGTAGTGTCGTCGGGATAC R: GCGTTTCTACCAAACAATTCG	LC green	143

**Table 3:** Direct comparison of DNA methylation markers in primary tumors and corresponding plasma cfDNA in advanced stage HGSOC (FIGO III). NA: non-available.

Patient ID	*BRCA1*		*SLFN11*		*USP44*	
	FFPEs	Plasma-cfDNA	FFPEs	Plasma-cfDNA	FFPEs	Plasma-cfDNA
#1	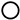	NA	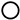	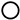	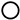	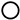
#2	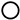	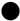	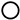	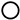	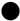	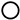
#4	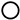	NA	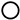	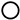	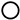	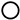
#6	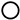	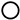	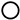	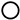	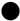	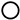
#8	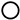	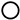	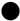	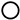	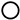	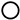
#9	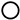	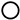	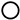	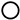	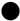	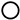
#10	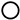	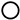	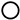	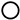	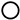	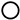
#15	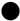	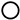	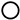	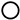	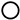	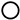
#17	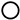	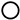	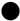	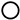	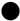	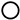
#18	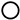	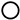	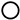	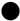	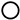	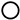
#19	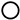	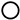	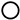	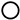	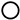	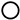
#20	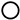	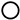	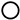	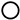	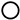	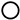
#22	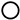	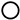	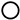	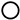	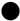	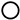
#23	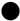	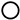	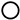	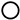	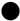	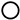
#24	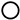	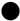	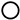	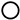	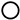	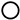
#26	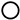	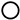	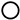	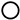	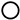	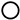
#27	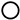	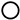	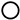	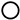	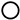	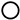
#29	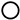	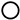	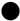	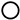	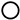	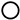
#32	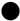	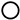	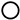	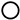	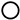	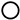
#33	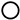	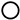	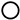	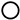	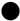	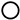
#34	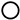	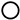	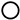	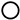	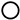	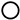
#38	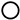	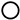	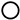	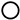	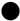	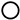
#39	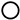	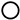	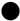	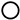	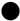	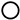
#40	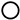	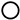	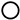	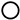	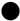	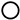
#41	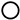	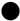	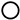	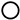	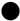	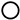
#43	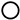	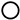	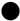	NA	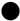	NA
#44	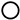	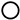	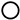	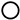	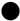	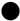
#45	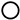	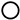	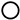	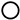	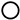	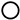
#47	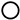	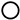	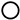	NA	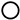	NA
#49	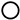	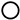	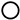	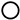	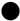	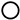
#50	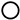	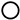	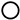	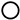	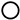	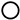
#54	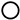	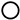	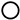	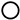	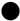	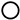
#55	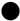	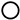	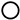	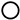	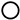	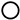
#56	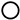	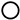	NA	NA	NA	NA
#57	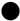	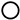	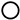	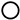	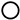	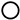
#58	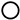	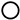	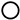	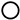	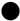	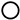
#59	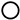	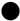	NA	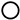	NA	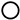
#60	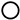	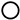	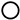	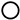	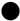	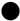
#61	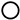	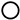	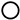	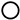	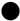	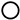
#62	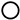	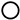	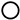	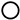	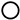	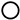
#63	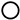	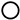	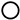	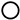	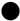	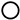
#64	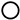	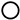	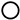	NA	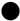	NA

**Table 4:** Direct comparison of DNA methylation of *BRCA1*, *SLFN11* and *USP44* gene promoters in primary tumors and paired plasma cfDNA in advanced HGSOC (FIGO III).

Primary Tumor	Paired Plasma cfDNA		
*BRCA1*	Unmethylated	Methylated	Total
Unmethylated	31	4	35
Methylated	5	0	5
Total	36	4	40
Agreement	31/40 (77.5%), *p* = 0.573, k = −0.125		
*SLFN11*	Unmethylated	Methylated	Total
Unmethylated	32	1	33
Methylated	4	0	4
Total	36	1	37
Agreement	32/37 (86.5%), *p* = 0.892, k = −0.045		
*USP44*	Unmethylated	Methylated	Total
Unmethylated	19	0	19
Methylated	16	2	18
Total	35	2	37
Agreement	21/37 (56.8%), *p* = 0.230, k = 0.114		

## Data Availability

The data presented in this study are available on request from the corresponding author. The data are not publicly available due to ethical restrictions.
